# A survey of accuracy of nurses’ clinical judgement of cutaneous graft‐versus‐host disease in Japan

**DOI:** 10.1002/nop2.669

**Published:** 2020-11-02

**Authors:** Rumi Maeda, Kyoko Obama, Akiko Tomioka, Junko Akagawa, Mitsue Maru

**Affiliations:** ^1^ Nursing Career Pathway Center Graduate School of Health Care Sciences Tokyo Medical and Dental University Tokyo Japan; ^2^ Division of Behavioral Sciences Research Center for Public Health Sciences National Cancer Center Tokyo Japan; ^3^ Division of Nursing Faculty of Healthcare Tokyo Healthcare University Tokyo Japan; ^4^ Tokyo Metropolitan Geriatric Medical Center Tokyo Japan; ^5^ Faculty of Nursing and Rehabilitation Department of Nursing Konan Women's University Kobe Japan

**Keywords:** care, clinical judgement, haematopoietic stem cell transplantation, nursing assessment

## Abstract

**Aim:**

We examined accuracy of nurses’ clinical judgement of graft‐versus‐host‐disease (GVHD) symptoms and related factors using Common Terminology Criteria for Adverse Events (CTCAE) for patients who developed chronic cutaneous GVHD after haematopoietic stem cell transplants.

**Design:**

Cross‐sectional design using nationwide survey.

**Methods:**

A questionnaire survey based on Tanner's clinical judgement model to assess patients with chronic cutaneous GVHD using CTCAE was used. Free‐text descriptions and statistical analyses of relationship between correct responses and demographic data were performed.

**Results:**

The rate of correct responses for main symptoms of skin GVHD was < 50%; there was no statistical significance between correct responses and demographic data, knowledge about GVHD and collaborative practice with physicians. The accuracy of cutaneous GVHD clinical judgements was not directly related to nurses’ background. Educational opportunities that reinforce nurses’ abilities to reflect on knowledge and experiences to interpret patient symptoms are essential for improving accuracy of clinical judgement.

## 1 INTRODUCTION

Graft‐versus‐host disease (GVHD) is a severe complication of haematopoietic stem cell transplantation (HSCT) that accounts for 25% of mortalities after HSCT (Svahn et al., 2012). Cutaneous symptom has been used as a monitoring indicator to decide treatment because it reflects the severity of systemic GVHD (Rodrigues et al., 2018; Ziemer, 2013). Nurses have many opportunities to observe patients’ skin symptoms through routine care. Nurses are expected to make accurate clinical judgements and to monitor symptoms of cutaneous GVHD to provide early detection, treatment and alleviation of symptoms with appropriate skin care. However, the accuracy of nurses’ clinical judgements of skin GVHD and related factors remains unclear.

## 2 BACKGROUND

According to the Worldwide Network for Blood and Marrow Transplantation, approximately 68,000 HSCTs are performed annually based on a global research study and the number of transplants has been increasing annually (Niederwieser et al., 2016). Among these, approximately 5,500 HSCTs are performed annually in Japan, ranking second after the United States in terms of frequency (The Japanese Data Center for Hematopoietic Cell Transplantation/The Japan Society for Hematopoietic Cell Transplantation, 2017).

The 5‐year survival rate after HSCT has increased to 53.2% with autologous transplantation (The Japanese Data Center for Hematopoietic Cell Transplantation/The Japan Society for Hematopoietic Cell Transplantation, 2019), although approximately 55% of transplant recipients develop GVHD (Rodrigues et al., 2018), thereby contributing to the decline in patients’ survival and prognoses as well as quality of life.

At present, there is no definitive treatment for GVHD; immunosuppressive drugs and skin care are used to alleviate symptoms. In addition, chronic skin GVHD symptoms may persist for several years, resulting in physical and psychological distress among patients (Yokota et al., 2011). The appropriate monitoring of skin symptoms by nurses is critical for the early detection and treatment of chronic GVHD and is also expected to contribute to the alleviation of skin symptoms through the implementation of appropriate skin care (Flowers & Martin, 2015). To detect/treat cutaneous GVHD early and choose appropriate skin care for alleviating symptoms, nurses’ accurate clinical judgements using multidisciplinary indicators are required.

**FIGURE 1:**
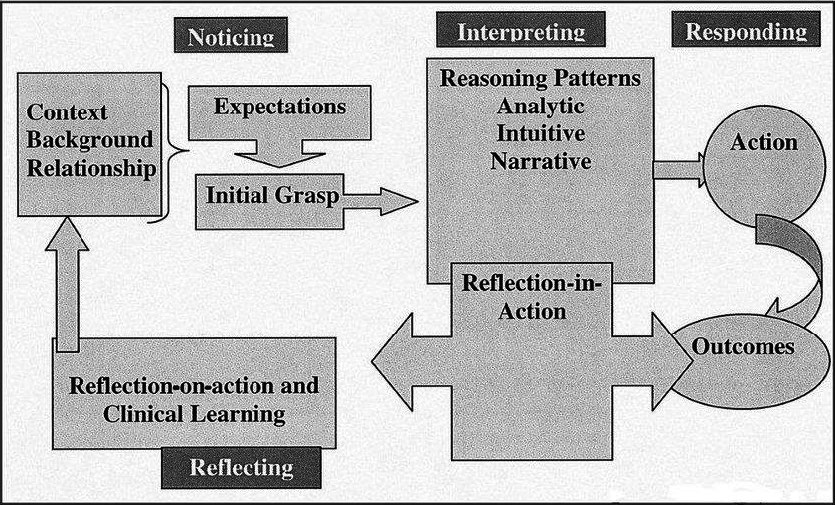
Clinical judgment model (Tanner, 2006)

The process of nurses’ clinical judgements was modelled by Tanner (Tanner, 2006); the model includes four aspects: “Noticing,” “Interpreting,” “Responding,” and “Reflecting” (Figure 1). According to this model, “Noticing” is the first aspect where skin symptoms and the status of patients are observed to gain an overall understanding of the situation. The next aspect is “Interpreting,” where symptoms are correlated to information such as clinical knowledge and the nurses’ own experiences to obtain sufficient understanding, followed by “Responding,” wherein clinical judgements and skin care method decisions are made. The final aspect is “Reflection,” wherein patients’ responses are observed, and a decision is made regarding whether or not the interpretation of information and judgement were correct. The model states that the nurses’ background, such as clinical knowledge and experiences, the political and social context, interdisciplinary relationships and disproportionate relationships, particularly with physicians, influence clinical judgements (Tanner, 2006).

One of the indicators to evaluate cutaneous GVHD is the Common Terminology Criteria for Adverse Events (CTCAE). CTCAE was developed at the National Cancer Institute and is recommended for use across multiple job functions (Japan Clinical Oncology Group [J.C.O.G., 2017]) as an indicator for evaluating unexpected symptoms associated with cancer treatment. CTCAE has also been used in symptom assessment in clinical studies and many studies have been conducted by nurses (Nagao et al., 2016; Oki et al., 2016; Yabuki et al., 2016). However, there have been no studies evaluating cutaneous GVHD using CTCAE.

### 2.1 Research question

This study aimed to: (a) determine the accuracy of nurses’ clinical judgements of skin symptoms using CTCAE for patients who developed chronic skin GVHD after HSCT; and (b) explore factors related to the accuracy of nurses’ clinical judgements of skin symptoms using CTCAE.

## 3 THE STUDY

### 3.1 Design

Cross‐sectional design using a nationwide postal and web survey and content analysis of free descriptions.

### 3.2 Methods

#### 3.2.1 Questionnaire development

**FIGURE 2:**
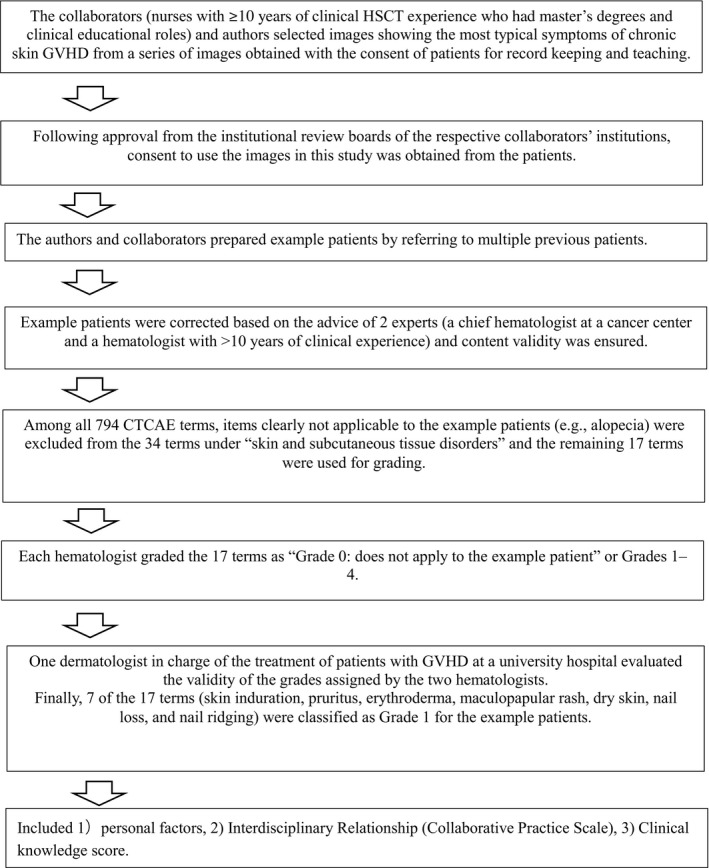
Questionnaire development process

**FIGURE 3:**
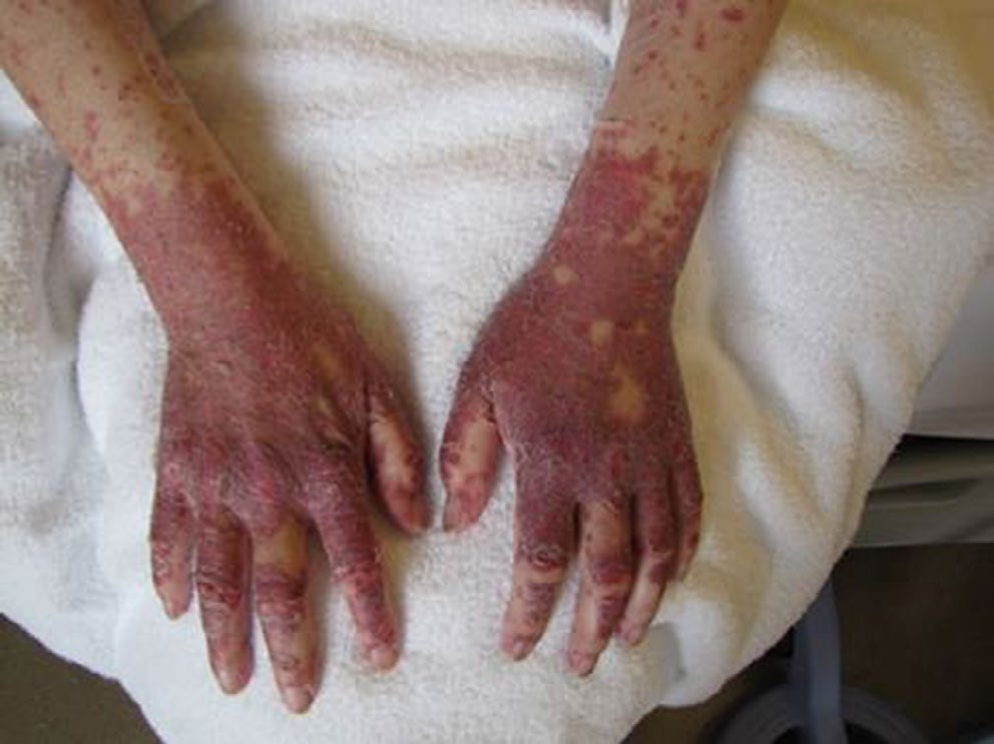
Summary of a chronic GVHD example patient and assessment of skin disorders using CTCAE. A female patient in her 40s and a housewife. Aplastic anemia observed 90 days after HLA‐identical unrelated allogeneic HSCT. *Disease history*: Engraftment was confirmed 7 days after transplantation, and the patient left the clean room on Day 40 after transplantation. The patient is currently hospitalized in the general ward. She presented with Grade 2 GVHD symptoms from Day 21 after transplantation and extensive skin induration from the upper arms to the fingers of both hands from approximately Day 80 after transplantation. Skin rupture of the wrist joint with exudate and skin desquamation were observed, which partially restricted her ADL. Symptoms were limited to the area shown in the image. No ointment or skin care has been used. At present, Prograf (graceptor) is being administered to the patient, the compliance with oral administration is good, and she is scheduled to be discharged from the hospital shortly. *Social background*: Family of 4 including a husband (40 years old), son (10 years old) and daughter (4 years old), and the patient’s mother is living in their neighborhood. The patient mentioned “being worried about returning home (with hands) looking like this,” and “her children getting scared upon seeing her hands,” and she appeared discouraged. CTCAE: Erythroderma Grade 1, dry skin Grade 1, nail loss Grade 1, nail ridging Grade 1, pruritus Grade 1, maculopapular rash Grade 1, skin induration Grade 1

The questionnaire development process is shown in Figure 2. The developed case is shown in Figure 3. The questionnaire asked the following questions; 1) Personal Factors measured included demographic data: (a) years of clinical experience; (b) years of HSCT nursing experience; (c) presence or absence of experience in caring for patients with cutaneous GVHD; (d) job role; (e) educational attainment; (f) use of CTCAE to assess cutaneous GVHD); and the “interdisciplinary relationships” was measured using the Japanese version of the Collaborative Practice Scales—Version for Nurses (hereinafter, CPS). The scale consists of a total of nine items, including two subscales for measuring the self‐assertiveness towards physicians, with four items on “expert knowledge and asserting opinions,” and five items for “clarifying each other's expectations of joint responsibilities.” This was evaluated on a six‐point Likert scale, the lowest score indicating “not practiced at all” (one point) to the highest score indicating “always practiced” (six points); the higher the total score, the more collaborative practice was carried out with physicians. Cronbach's α for the Japanese version of this scale was reported to be 0.92 and it has been confirmed that the Japanese version of CPS for nurses was consistent with the original version. The Japanese version of CPS for nurses’ reliability and validity has been ensured (Komi et al., 2010).

3) The clinical knowledge test regarding cutaneous GVHD was internally prepared via the following procedures. Fifty questions were prepared to obtain knowledge on the pathophysiology of GVHD, skin assessments and skin care with reference to the 51 questions on GVHD and skin care from the “Clinical and educational ladder for nurse to be engaged in haematological cancer nursing including HSCT” created by The Japan Society for Hematopoietic Cell Transplantation. The questions were reviewed by two haematologists, one dermatologist (an expert in cutaneous GVHD treatment), one clinical nurse with a master's degree and nursing experience in HSCT patients and one clinical nurse with a master's degree in nursing to investigate content validity. A total of 25 questions that may be prioritized as knowledge that nurses should have was selected by these internal experts. Questions were written in the form of “yes” “no” questions. Correct answers scored 1 point each for a maximum score of 25 points.

#### 3.2.2 Questionnaire distribution

Paper‐ and web‐based questionnaires were prepared to record symptom assessments using free text. Both paper‐ and web‐based questionnaires included same questions and the participants could choose either one as per their convenience. Questionnaire pretesting was performed on a total of six participants, two of whom were nurses with HSCT nursing experience (one was a certified nurse specialist [CNS] in oncology nursing) and four without such experience, to ensure face validity.

Questionnaires were sent to target participants using the following methods. Registered nurses (RNs) were sent questionnaires by mail to the directors of the nursing departments of the target sites along with a document explaining the purpose of the study and an access method manual for the web‐based questionnaire. CNSs and certified nurses (CNs) were sent questionnaires directly to their prospective sites with a document explaining the purpose. The participants responded by returning the questionnaire or submitting responses online for paper‐ and web‐based questionnaires. Submitted responses were considered consent to participate in the study.

#### 3.2.3 Participants

The paper‐ or web‐based questionnaires that included the same questions were distributed to a total of 3,022 participants, including 2,056 nurses working in wards or outpatient settings and 966 CNs and CNSs. A total of 237 responses (recovery rate: 7.8%) were received, with 23 via the web and 214 via mail. Among these, 216 responses were included after disqualifying those without ≥ 1 CTCAE terms in the analyses (valid response rate: 91.1%). (a) Nurses working at Japan Marrow Donor Program transplant‐certified sites (218 wards at 168 sites) in wards or outpatient settings administering post‐HSCT care to patients; and (b) CNSs in oncology nursing; CNSs in child health nursing; CNs in wound, ostomy and continence nursing; and CNs in chemotherapy nursing working at institutions mentioned in the Japanese Nursing Association website were included.

Japanese CNSs are advanced practice nurses who have a master's degree. CNs are those who have obtained qualifications after completing training for 6 months at a training centre certified by the Japanese Nursing Association (Japanese Nursing Association, 2016). There were no restrictions on age, sex, number of years of nursing experience, or type of employment.

#### 3.2.4 Survey period

October–December 2015.

### 3.3 Analysis

#### 3.3.1 Statistical analysis

To verify if the CTCAE terms corresponding to events related to example patients were correctly selected, when “Grade 1 or higher” (symptoms present) was selected for the seven terms corresponding to events related to example patients or when Grade 0 (without symptoms) was selected for the terms not corresponding to events related to example patients, 1 point was given; on the other hand, responses not fitting the above conditions were given 0 points and scores were obtained for each response (CTCAE scores).

Consequently, CTCAE scores were correlated with the number of years of nursing experience, CPS and the clinical knowledge test. In addition, *t*‐tests were performed for each pair of nurses with/without CN/CNS qualifications, nurses with/without experience in providing care for patients with GVHD and using or not using CTCAE for assessment.

Subsequently, to determine whether there was a significant difference in the number of correct responses among the CTCAE terms and if responses of Grade 1 or higher were given for the seven terms corresponding to events related to example patients, the frequency of responses of Grade 0 among the 10 terms not corresponding to events related to example patients was obtained. Thereafter, the Friedman test was performed to assess the difference between events. When significant differences were found, pairwise comparisons were performed with multiple comparisons and Bonferroni's correction.

Statistical tests were performed using SPSS Statistics Base ver. 26 and *p* < .05 was considered statistically significant. Priori analyses were performed using G*Power 3.1. The required sample size for G*Power 3.1 was 136 (effective size: 0.4, power: 0.8).

#### 3.3.2 Comparison of free‐text entries

To clarify the differences in skin assessment, the content of free‐text entries was analysed using methods based on content analysis by Krippendorff. A group with higher CTCAE scores (higher CTCAE score group) and another with lower CTCAE scores (lower CTCAE score group) were created and free‐text entries regarding each skin assessment were used as raw data, with one sentence or each related sentence regarded as one unit and coded. Codes were summarized and classified based on similarities and differences in semantic content, and categories were created. The categories and codes in the study groups were compared. The validity of the classification of free‐text entries was evaluated by four nursing researchers. Krippendorff's alpha was 0.91.

### 3.4 Ethics

This study was conducted in accordance with the Declaration of Helsinki, approved by the institutional review boards of Shizuoka Cancer Center (approval number: T25‐40‐25‐1) and the Faculty of Medicine of Tokyo Medical and Dental University (No. 1636).

## 4 RESULTS

### 4.1 Response status and participant attributes

The questionnaires were distributed to 3,022 participants including 2,056 nurses working in wards or outpatient settings and 966 CNs and CNSs. Overall, 237 responses (23 and 214 via web and postal mail, respectively; recovery rate, 7.8%) were received. Among these, 216 responses were included after disqualifying those without ≥ 1 CTCAE terms in the analyses (valid response rate: 91.1%).

The participants were nurses with an experience of 11.8 (*SD* = 7.0) years (range: 1–37, mode: 11) and HSCT nursing experience of 5.4 (*SD* = 4.0) (range: 0–20, mode: 5) and they included 158 RNs (74.9%) (Table 1). The most common department was the adult ward (76 participants, 30.0%). The allogeneic transplant number per year at affiliated institutions was the highest for 103 participants (47.7%), with 57 (26.4%) having performed ≥ 20 transplants annually.

**TABLE 1:** Clinical judgement of cutaneous graft‐versus‐host disease survey participant attribution (*n* = 216)

Attribution	Number of participants	%
Gender		
Female	201	93.1
Male	11	5.1
No response	4	0.8
Education		
Diploma	117	54.2
Bachelor	72	33.3
≥Master	19	8.8
Other	5	2.3
No response	3	1.4
Occupation		
Registered nurse	158	74.9
Certified nurse in cancer chemotherapy	21	8.3
Certified nurse in wound, ostomy and continence nursing	18	7.1
Certified nurse specialist in oncology nursing	10	4.0
Certified nurse specialist in child health nursing	4	1.6
No response	5	2.0

### 4.2 Providing care and CTCAE use status

Approximately 50% participants had experience of providing care to patients with chronic skin GVHD. Of these, 23.6% responded with “symptoms assessed using CTCAE for skin disorders” (Table 2). There were no significant differences in CTCAE use between CNSs/CNs and RNs.

**TABLE 2:** Clinical judgement of cutaneous graft‐versus‐host disease survey institutional attribution (*n* = 216)

Attribution	Number of participants	%
Department		
Adult ward	76	30.0
Paediatric ward	35	13.8
Clean room	26	10.3
Outpatient	19	7.5
Mixed adult and paediatric ward	16	6.3
Other	38	17.6
No response	6	14.5
Annual number of allogeneic transplants (total)		
<10	103	47.7
10–20	49	22.7
≥20	57	26.4
No response	7	3.2
Experience providing care to patients with chronic skin GVHD		
Yes	111	51.4
No	103	47.7
No response	2	0.9
Status of the use of CTCAE		
Prior knowledge and use in assessments	77	35.6
Prior knowledge but do not use in assessments	61	28.2
Unaware	78	36.1
Symptoms assessed using CTCAE	(Duplicate responses) *n* = 77	
Oral mucosal disorder	64	83.1 (29.6)
Gastrointestinal symptoms	56	72.7 (25.9)
Skin disorders	51	66.2 (23.6)
Other	9	11.7 (4.2)

Note

Percentages of the total (*n* = 216) displayed in parentheses

### 4.3 Relationship between CTCAE scores and nursing experience or advanced practice nursing qualifications

The mean CTCAE score was 11.9 (*SD* = 2.05) points (range: 6–17) of a total of 17 points and the mean score when converted to a total of 100 points was 69.9 (*SD* = 12.1) points.

There was no significant correlation between CTCAE scores and number of years of nursing experience (*r* = 0.069, *p* = .314) and between CTCAE scores and number of years of transplant nursing experience (*r* = 0.132, *p* = .055).

Similarly, there were no significant differences in CTCAE scores based on *t*‐tests between the presence and absence of experience of providing care to patients with chronic skin GVHD [t(203) = 1.711, *p* = .089], between RNs and CNSs/CNs [t(209) = 0.735, *p* = .463] and between use and non‐use of CTCAE as an indicator of skin disorders [t(214) = 1.267, *p* = .207)].

### 4.4 Correlations among knowledge test, collaborative practice scales and CTCAE scores

The mean score of the knowledge test was 18.20 of 25 points [*SD* = 2.14, range 11–23] and the mean total CPS score was 3.49 of 6 points (*SD* ± 1.06, range 1.0–5.67). The mean score for the “expert knowledge and asserting opinions” subscale was 3.85 of 6 points (*SD* = 1.15, range 1.0–6.0) and that for the “clarifying each other's expectations of joint responsibilities” subscale was 3.20 of 6 points (*SD* = 1.11, range 1.0–5.4). Mean scores on the subscales were above those of the Japanese version reported by Komi et al., (2010) (total score 2.74 *SD* = 1.03 points, “expert knowledge and asserting opinions” subscale 3.09 *SD* = 1.18 points and “clarifying each other's expectations of joint responsibilities” subscale 2.46 *SD* = 1.03 points). Correlations among CTCAE scores, CPS and knowledge tests were *r* < 0.1 (*p* > .05).

### 4.5 Accuracy of CTCAE assessment of skin disorders and determination of severity

Differences between terms were observed based on Friedman test results (test statistic = 1,200.221, DF = 16, *p* < .001). Terms showing significant differences in multiple comparisons (*p* < .005) are summarized in Table 3. The rate of correct responses for maculopapular rash (monitoring index for chronic skin GVHD) was ≤ 50% and there were many correct responses with significant differences. The rate of correct responses for symptoms presenting with skin discoloration like erythema multiforme and purpura was ≤ 50% and compared with other terms, there were many correct responses with significant differences.

**TABLE 3:** Rate of correct responses in CTCAE assessments (*n* = 216)

Term	Rate of correct responses (%)	Results of multiple comparisons (*p* < .005 terms)
**Dry skin**	**95.8**	**17**
Urticaria	94.0	12
Toxic epidermal necrolysis	93.1	12
**Skin induration**	**84.3**	**17**
Telangiectasia	82.9	11
Bullous dermatitis	81.9	**11**
**Pruritus**	**81.9**	**17**
Stevens–Johnson syndrome	77.8	11
Skin ulceration	72.7	12
Acneiform rash	70.8	7
**Nail loss**	**60.6**	**14**
**Nail ridging**	**58.3**	**15**
**Erythroderma**	**55.6**	**16**
Nail discoloration	48.1	10
Purpura	44.4	15
Erythema multiforme	43.1	17
**Maculopapular rash**	**42.6**	**14**

Note

**Text in bold** indicates symptoms in example patients

### 4.6 Accuracy of CTCAE grading

The frequency of each grade for the seven terms including symptoms of chronic GVHD is shown in Table 3. The rate of correct responses for all terms was < 60% and that for maculopapular rash and erythroderma (monitoring indices) was only 10%–20% (Table 4).

**TABLE 4:** Response distributions for CTCAE grading of example patients (*n* = 216)

Chronic GVHD	Frequency	%
Dry skin*		
Not applicable	9	3.6
**Covering < 10% body surface area (BSA)**	**26**	**10.3**
Covering 10%–30% BSA	133	61.6
Covering > 30% BSA	48	22.2
Erythroderma*		
Not applicable	96	44.4
**Covering > 90% BSA without associated symptoms**	**49**	**22.7**
Covering > 90% BSA with associated symptoms	70	32.4
Covering > 90% BSA with associated fluid or electrolyte abnormalities	1	0.5
Nail loss		
Not applicable	85	39.4
**Asymptomatic separation of the nail bed from the nail plate or nail loss**	**106**	**49.1**
Symptomatic separation of the nail bed from the nail plate or nail loss	25	11.6
Nail ridging		
Not applicable	90	41.4
**Asymptomatic or intervention not indicated**	**126**	**58.3**
Pruritus*		
Not applicable	39	18.1
**Mild or localized**	**136**	**63.0**
Intense or widespread or intermittent	35	16.2
Intense or widespread or constant	6	2.8
Maculopapular rash*		
Not applicable	124	57.4
**covering < 10% BSA**	**25**	**11.6**
covering 10%–30% BSA	52	24.1
covering > 30% BSA	15	6.9
Skin induration*		
Not applicable	34	15.7
**Mild induration**	**72**	**33.3**
Moderate induration	71	32.9
Severe induration	39	18.1

Note

**Text in bold** indicates correct responses (grade for the example patient)

* Items in which “degree to which symptoms affect activities of daily living (ADL)” was used as criterion for grading

### 4.7 Free‐text entry of assessments

Responses of 27 and 43 participants in the lower and higher (6–9 and 14–17 points, correct responses: 35.3%–52.9% and 82.4%–100%), respectively, were extracted. There were no significant differences between these groups in terms of the codes for “dry skin/epidermolysis,” “scleroderma,” and “change in skin colour,” which are symptoms of chronic skin GVHD. However, regarding the code for “skin care based on the assessment,” the higher CTCAE score group indicated the skin care purpose as a means “to not exacerbate symptoms” and the content of skin care was listed in detail according to symptoms like dryness and nail protection. The entered content in the lower CTCAE score group did not specifically describe some characteristics such as affected activities of daily living (ADL) and there was no specificity in the content of skin care based on skin assessment (Table 5).

**TABLE 5:** Examples of free‐text entries in the higher CTCAE score group (*n* = 43)

The skin of the finger joint is broken, there is exudate, the defence mechanism of the skin is dysfunctional, and the skin is in an infection‐prone condition. In addition, finger numbness and skin tightness in the wrist interferes with activities of daily life (ADL). The attending physician is considering discharge. Patients understand the benefit of Prograf and agree to continue the drug. However, patients seem to be anxious about ADL/IADL restrictions due to the present state of his/her fingers and the reactions of his/her family to the skin condition. Skin tightness and scleroderma‐like symptoms are present. There is a need to start performing sufficient skin care because it is not being performed. It is necessary to promote skin softening. Is there speculation of whether patients can take the tablet by themselves (take the tablet and consume)? Is it necessary to administer medical treatments such as ointments other than oral medications? Is there anything that can be used to treat itching? → There is a need to confirm the necessity of medical measures for skin symptoms and to give guidance on self‐care for daily life.

## 5 DISCUSSION

The recovery rate for the study questionnaire was low at 7.8% (effective response rate: 91.1%). Since the enactment of the Act on the Protection of Personal Information in 2003, a decline has been observed in the recovery rate of questionnaires via mail in Japan (Go, Hiroyuki, & Satoshi, 2006), with the highest rate estimated to be 20% (Hayashi, 2016). This survey showed that the institution type, number of transplants performed, basic educational history of participants, sex and care history of patients with skin GVHD are adequately reflected among nurses employed at HSCT sites in Japan. The required number of samples was achieved to obtain sufficient statistical power analysis.

### 5.1 Differential diagnosis of chronic skin GVHD and grading with CTCAE

The grading of characteristic symptoms such as maculopapular rash and skin induration are considered based on “effects on daily life,” which is roughly classified under “instrumental ADL,” and “self‐care ADL,”; however, clear criteria have not been specified even in CTCAE ver. 5.0. There is a possibility that these weak points may be related to the low CTCAE score. In this study, the rate of selection for main symptoms of skin GVHD, that is maculopapular rash and erythroderma, were < 50%. Approximately 75% of nurses could not correctly differentiate between the main symptoms and erythema multiforme/purpura. The accuracy of the assessment of skin GVHD did not merely on experience in providing care or advanced practice nursing qualifications. A previous study (Peuvrel et al., 2018) identified: “(a) the selection of appropriate symptom categories; and (b) assigning grade” as concerns in assessing skin symptoms using CTCAE. The accuracy of assessments using CTCAE is also important to improve (c) the accuracy of differential diagnoses for skin eruptions and diseases associated with skin colour changes.

On the other hand, in the free‐text entries on assessments of subjective skin symptoms, such as itchiness, the higher CTCAE score group described many free‐text entries of such symptoms in relation to specific ADL and the hypothesis is that an assessment based on this relationship leads to accurate differential diagnoses. In this study, nurses with higher scores made an evaluation of concrete skin care methods based on patient ADL in addition to subjective symptoms with the objectives of “preventing the lowering of ADL.” ADL of patients with skin GVHD rely on hands and joint mobility, which has a close relationship with subjective symptoms of skin GVHD. There is a possibility that nurses who gave higher scores diagnosed subjective skin symptoms more carefully to choose appropriate skin care methods for preventing the lowering of ADL and this may help in grading CTCAE more accurately.

### 5.2 Factors related to clinical judgement

CTCAE scores were not associated with all the knowledge listed in the CJM, collaborative practice with physicians, years of nursing experience, experience with participating in study meetings, or care experience.

This result might be attributed to the fact that 63.8% of all participants knew about CTCAE, yet only 23.6% used it for the assessment of skin disorders. In this study, 47.7% of participants worked at institutions that performed <10 transplants/year, which indicates a lack of opportunities for CTCAE use; thus, the low CTCAE scores.

There have been few research reports to date on the association between nurses’ clinical judgements and collaborative practice with other job functions. The current survey used case studies; thus, it is likely that the direct association between collaborative practice with physicians and nurses’ ability to make clinical judgements could not be measured. However, the total CPS score and subscale scores of participants in this survey were higher than the mean for the Japanese version reported by Komi et al. (2010) and “disproportionate relationships with physicians” that affected clinical judgements were minimal.

Interestingly, in the current survey, clinical knowledge and experiences, which are believed to improve clinical judgements, were also not directly related to CTCAE scores. According to the CJM, grasping the situation by “Noticing,” then relating and analysing the knowledge and information in the process of “Interpreting” are important aspects to ensure accurate judgements are made (Tanner, 2006). In the higher CTCAE score group, a large number of free‐text statements about ADL were given and these analyses may have helped not only in the selection of skin care methods but also in grading CTCAE as a narrative analysis in the CJM. The lack of direct correlations between collaborative relationships with physicians, clinical knowledge and nurses’ experiences with clinical judgements supported the CJM concept that “background factors” such as clinical knowledge and experiences do not translate into correct clinical judgements if the appropriate interpretation is not made. Various educational interventions using CJM are now being considered (Nielsen, 2016; Timbrell, 2017); educational interventions to improve the clinical judgement of cutaneous GVHD using CTCAE are essential.

### 5.3 Limitations

Although the target participants seemed to reflect the actual conditions of nurses who work at HSCT sites in Japan, based on the low recovery rate, the possibility that only nurses who had a high interest in skin GVHD actively responded cannot be excluded.

### 5.4 Conclusion

- Nurses were not able to discriminate and grade key symptoms that serve as monitoring indicators for clinical judgements using CTCAE for cutaneous GVHD.
- The accuracy of cutaneous GVHD clinical judgements was not directly related to nurses’ background.
- To make accurate judgements, the mastery of experiences and knowledge and the process of “Interpreting,” wherein knowledge is correlated to patient information and appropriate analyses are performed, are crucial.
- Continuing education opportunities to increase the accuracy of interpretation and improve clinical judgements are needed.

## CONFLICT OF INTEREST

None of the authors have any conflicts of interest or any financial ties to disclose.

## Data Availability

The data that support the findings of this study are available on request from the corresponding author. The data are not publicly available due to restrictions, for example their containing information that could compromise the privacy of research participants.
